# Impact of quantified knee positioning on the measurement of minimal joint space width using statistical shape models: A cross-sectional and longitudinal analysis in the IMI-APPROACH^☆^

**DOI:** 10.1016/j.ostima.2025.100357

**Published:** 2025-06-25

**Authors:** Eva A. Bax, H. Chien Nguyen, Roel J.H. Custers, Vahid Arbabi, Hassan Rayegan, Willem Paul Gielis, Claudia Lindner, Tim F. Cootes, Margreet Kloppenburg, Francisco J Blanco, Ida K. Haugen, Francis Berenbaum, Mylène P. Jansen, Simon C. Mastbergen, Frank W. Roemer, Felix Eckstein, Wolfgang Wirth, Moyo C. Kruyt, Harrie Weinans

**Affiliations:** a Department of Orthopaedic Surgery, University Medical Center Utrecht, the Netherlands; b 3D Lab, University Medical Center Utrecht, the Netherlands; c Orthopedic and Biomechanical Research Group, University of Birjand, Iran; d Division of Informatics, Imaging and Data Sciences, The University of Manchester, United Kingdom; e Departments of Rheumatology, Clinical Epidemiology, Leiden University Medical Center, the Netherlands; f Grupo de Investigación de Reumatologia (GIR), INIBIC-Complejo Hospitalario Universitario de A Coruña, Centro Interdisciplinar de Quimica y Biologia, CICA-UDC, Universidad de A Coruña, Spain; g Center for Treatment of Rheumatic and Musculoskeletal Diseases (REMEDY), Diakonhjemmet Hospital, Norway; h Department of Rheumatology, Sorbonne University, INSERM CRSA, AP-HP Saint-Antoine Hospital, France; i Department of Rheumatology & Clinical Immunology, University Medical Center Utrecht, the Netherlands; j Department of Radiology, Universitätsklinikum Erlangen & Friedrich-Alexander-Universität Erlangen-Nürnberg (FAU), Germany; k Department of Radiology, Boston University Chobanian & Avedisian School of Medicine, USA; l Research Program for Musculoskeletal Imaging, Center for Anatomy & Cell Biology and Ludwig Boltzmann Institute for Arthritis & Rehabilitation, Paracelsus Medical University Salzburg, Austria; m Chondrometrics GmbH, Germany; n Department of Biomechanical Engineering, Faculty 3mE, TU Delft, the Netherlands; o Department of Mechanical Engineering, Bozorgmehr University of Qaenat, Iran

**Keywords:** Knee positioning, Minimum joint space width, Statistical shape models, Cartilage thickness, Knee osteoarthritis

## Abstract

**Objective:**

Correlations between minimum joint space width (mJSW) and MRI-based cartilage thickness are strong in cross-sectional analyses and moderate in longitudinal analyses, possibly due to knee rotation and flexion. This study investigates the effect of knee positioning during radiographic acquisition on the difference between mJSW and MRI-based cartilage thickness.

**Methods:**

Radiographic mJSW from the index knee was determined from baseline (265 patients) and 24-month follow-up (165 patients) on fixed-flexion radiographs from IMI-APPROACH (multicenter OA study) patients using automated software. Statistical Shape Models were used to quantify knee rotation and flexion on radiographs. Cartilage thickness was assessed by manual segmentation from MRI. Differences between mJSW (radiographs) and cartilage thickness (MRI) were assessed at baseline and follow-up. Multivariable linear regression was used to evaluate the impact of knee flexion and rotation on the difference between mJSW and cartilage thickness.

**Results:**

In cross-sectional analysis, differences between X-ray and MRI were significantly influenced by knee rotation (β = -0.18, P < 0.001). Longitudinal change in differences between X-ray and MRI were associated with changes in knee flexion (β = 0.19, P=0.002). Increases of one standard deviation in internal rotation and extension at follow-up resulted in a 0.2 mm false surrogate measurement of cartilage changes on radiographs.

**Conclusion:**

Quantified knee positioning significantly affects differences between mJSW measured on radiographs and MRI-based cartilage thickness. The longitudinal analyses revealed that knee flexion was related to these differences, while knee rotation was only related to cross-sectional differences. These findings highlight the importance of knee positioning during radiographic acquisition in contributing to false surrogate measurement of cartilage status and cartilage change.

## 1 Introduction

Radiographic evaluation is a widely used method for diagnosing and monitoring the severity and progression of knee osteoarthritis (OA) [1,2]. The minimum joint space width (mJSW) measured on a posteroanterior knee radiograph in semi-flexed joint position under loading is commonly used as a surrogate measure of cartilage and meniscus integrity and/or degeneration, with the intent to monitor progression of knee OA [[2], [3], [4], [5]]. The JSW is integrated into widely adopted categorical grading systems that try to capture knee OA severity on radiographs, such as the Kellgren & Lawrence (KL) score [[5], [6], [7], [8]]. However, the KL score is less sensitive to subtle changes in quantitative parameters, such as changes in the continuous measure of mJSW [2]. Accurate and reliable measurement of radiographic OA parameters are essential for assessing structural changes in knee OA, especially when evaluating the effectiveness of new treatments [9].

Radiographic JSW reflects not only cartilage thickness, but is also influenced by additional factors, such as the meniscus and positional differences during aquisition [[10], [11], [12], [13]]. While cross-sectional studies often reveal strong correlations between JSW and cartilage thickness measurements obtained from non-weight-bearing Magnetic Resonance Imaging (MRI) [14,15], longitudinal analyses demonstrate only moderate correlations between changes in JSW and cartilage thickness [[16], [17], [18]]. Possible explanations for these differences include positional differences during radiographic acquisition, changes in meniscal extrusion and lower cartilage quality [19]. Variations in knee rotation and flexion are well-recognized limitations in assessing knee OA progression when using radiographs [12,13,[20], [21], [22]]. For instance, Kan et al*.* [12] reported significantly smaller medial JSW in flexion views compared to extended views, while Kinds et al*.* [13] observed that full leg extension increased JSW measurements.

Positional differences during radiographic acquisition, such as knee rotation and flexion, can be captured with statistical shape models (SSM). SSMs describe bone shapes observed in radiographs, capturing both anatomical differences (e.g., wider condyles) and positioning effects (e.g., knee rotation or flexion). The SSMs quantify subtle variations in knee positioning on radiographs. Shape variations are categorized into distinct shape modes, allowing separation between true anatomical characteristics and those due to patient positioning [[23], [24], [25], [26]]. Hence, if sequential radiographs are being made in time and no large changes in bone geometry are expected, the SSM will mostly represent the positioning with respect to radiographic acquisition. The aim of this study was twofold. Firstly, to determine whether cross-sectional differences between radiographic based mJSW and MRI-based cartilage thickness can be attributed to positioning during radiographic acquisition. Secondly, to evaluate the effect of change of positioning during radiographic acquisition over time. This longitudinal analysis provides insights into the impact of positioning on the thoughtfulness of progressors and non-progressors of KOA. We hypothesized that differences in positioning of knee flexion and rotation during radiographic acquisition will bias the interpretation of measured mJSW (surrogate) when compared to MRI-based cartilage thickness.

## 2 Methods

### 2.1 Patients

The prospective Applied Public-Private Research enabling OsteoArthritis Clinical Headway (IMI-APPROACH cohort) cohort is an observational, longitudinal cohort that enrolled patients with predominantly femorotibial OA selected from five European cohorts [27] ((CHECK (Utrecht, The Netherlands), HOSTAS (Leiden, The Netherlands), MUST (Oslo, Norway), PROCOAC (A Coruña, Spain), and DIGICOD (Paris, France)) or from their outpatient departments. Recruitment relied on machine-learning models to predict the probability of increased or sustained knee pain or structural progression during the two-year follow-up [27]. The index knee of the participants was selected based on American College of Rheumatism (ACR) criteria. If both knees met the ACR criteria, participants indicated their most affected knee to use as index knee; if participants indicated no difference, the right knee was chosen as index knee. A detailed description of the inclusion and exclusion criteria has been published previously [27]. Medical ethics committees of all participating centers approved the study, and all patients provided written informed consent. The study was registered under clinicaltrials.gov nr: NCT03883568.

### 2.2 Imaging protocol for knee radiography and MRI

Knee radiographs and MRI-scans of the patients were included in this study. The radiographs were obtained following the Buckland-Wright protocol, with a posteroanterior view of the knee in semi-flexed position (7${}^{\circ }$ - 10$7^{\circ }-10^{\circ }$) and under weight-bearing conditions [3,27]. The MRI protocol included sagittal 3D spoiled or volume-interpolated gradient echo sequences, incorporating selective water excitation or fat suppression techniques for quantitative cartilage morphometry. These 3D MRI scans were independent of patients positioning. Furthermore, sagittal, axial, and coronal intermediate-weighted fat-suppressed sequences were acquired for the assessment of the MRI Osteoarthritis Knee Scores (MOAKS). Among the five participating centers, two centers used 1.5T scanners, while the remaining three centers deployed 3T systems.

### 2.3 Imaging assessment

This study included knee radiographs of the index knee at baseline and 24 months follow-up. The radiographic mJSW (mm) in the medial compartment was provided by automated software (Orthopedic Digital Image Analysis (ODIA)) [28]. The KL score (range: 0-4) was determined at both time points by one experienced reader [27].

The medial femorotibial compartment cartilage thickness (mm) of the index knee was determined on MRI-scans at baseline and 24-months follow-up. Cartilage thickness was manually segmented from the MRIs by experienced readers using custom software (Chondrometrics, GmbH, Freilassing, Germany). This involved measuring the mean cartilage thickness (mm) of the medial tibia (MT) and the weight-bearing (central) part of the medial femur (cMF). The cartilage thickness for the weight-bearing medial femorotibial compartment (MFTC) was calculated (MFTC = MT + cMF) [29]. The quantitative cartilage measurements performed on MRI were used in this study as a reference (ground truth) for the surrogate cartilage thickness of mJSW measurements on radiographs, thereby including the meniscus status (based on MRI) as relevant parameter for radiographical mJSW.

Meniscal extrusion and meniscus damage were assessed using the semi-quantitative MOAKS instrument by an experienced radiologist blinded to clinical data, as meniscal extrusion and damage also influences the mJSW ^30^. In both the medial and lateral compartment, medial and lateral meniscal extrusion was scored. Each score ranges between 0-3: Grade 0: <2mm; Grade 1: 2 to 2.9mm, Grade 2: 3-4.9mm; Grade 3: >5mm [30]. The presence of meniscal extrusion was defined as a displacement of more than 3 mm of the medial or lateral meniscus [31]. Meniscal damage of the medial and lateral body was classified on a scale from 0 to 8, with grades 0 and 1 indicating an intact meniscus without a tear. Grades 2 to 4 correspond to varying types of tears, while grades 6 to 8 reflect maceration, signifying loss of meniscal substance [30]. A distinction was made between an intact meniscus (grades 0 and 1) and a non-intact meniscus (all other grades).

### 2.4 Statistical shape modelling

**Fig. 1:**
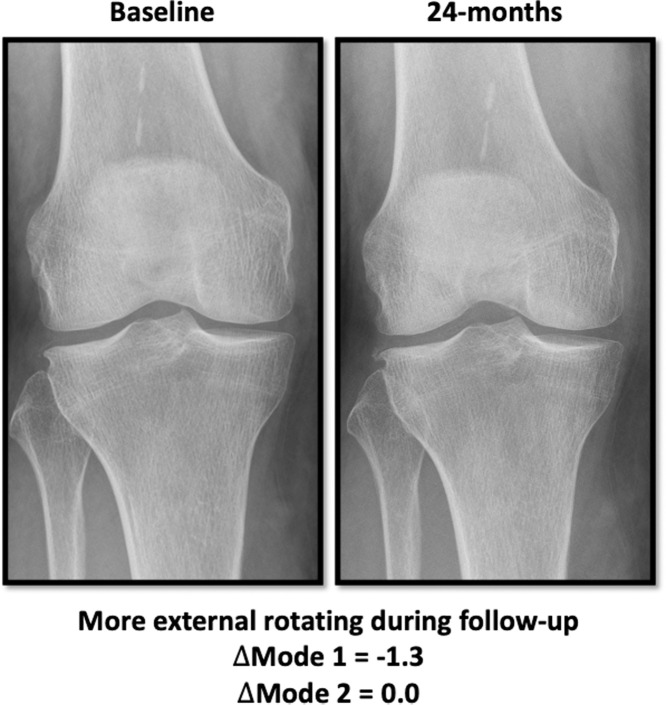
Example of knee radiographs of the same patient at baseline (left) and 24-month follow-up (right). At follow-up, the knee is positioned in greater external rotation, as indicated by a change in shape mode 1 (ΔMode 1 = –1.3), while the degree of flexion/extension remains unchanged (ΔMode 2 = 0.0). SSMs allow for the quantification of such subtle differences in knee positioning, which are often not visually apparent.

We used the validated software BoneFinder® (www.bone-finder.com, The University of Manchester, UK) to automatically annotate landmarks on knee radiographs [[32], [33], [34]]. The landmarks included the edges of the distal femur, proximal tibia, and patella [28]. These annotated landmarks served as input for generating the SSM based on a principal component analysis (PCA) to identify the predominant factors responsible for the observed variations in bone morphology. PCA simplified the data, generating independent modes of shape that reflect the variation from the average shape [26]. Each shape mode was quantified in standard deviations. As knee flexion and rotation with respect to the radiographic source and detector is also represented in the 2D projection of the radiograph, this positioning is also revealed in the SSM. SSMs can quantify subtle variations in knee positioning that are not always visually apparent (Fig. 1), by comparing each individual radiograph to the average knee positioning of the cohort. In this way, the SSM serves as a measurement method to quantify positioning variations. SSM was generated from knee radiographs of the index knee at baseline and at 24-month follow-up. Shape analyses of the IMI-APPROACH cohort revealed that the two largest principal components indicate knee rotation (shape mode 1) and knee flexion (shape mode 2), both expressed in standard deviations (SD).

### 2.5 Design of the study

This study consists of two phases, using patients from the IMI-APPROACH cohort. In the first phase, a cross-sectional analysis assessed whether differences between mJSW and MRI-based cartilage thickness can be attributed to knee flexion and rotation during acquisition, with the MRI-based meniscus status considered. All patients with baseline radiographs and MRI scans of the index knee were included. In the second phase, a longitudinal analysis was performed, calculating the difference in knee flexion and rotation over 24 months (∆Mode 1 and ∆Mode 2) and the changes in mJSW (∆mJSW) and MRI cartilage thickness (∆Cartilage thickness) over 24 months, incorporating MRI-based meniscus status as a parameter. The longitudinal ∆difference was determined by subtracting ∆mJSW from ∆Cartilage thickness, which provided the difference in MRI-based and radiographical-based change in mJSW. Patients with both baseline and 24-month radiographs and MRI scans were included. For the longitudinal analysis, baseline meniscal extrusion and meniscal damage were used.

### 2.6 Statistical analysis

All statistical analyses were performed using Statistical Package for the Social Sciences (SPSS) version 27.0 software (IBM, Armonk, New York). Descriptive statistics, displaying means and standard deviations (SDs), or numbers and percentages, were provided. Histograms were assessed for normal distribution. Univariable linear regression was employed to assess the impact of Mode 1 (internal/external rotation), Mode 2 (flexion/extension), meniscal extrusion, and meniscal tears on the difference between MRI-based cartilage thickness and minimal joint space width (mJSW) independently. Moreover, multivariable linear regression was used to analyze the impact of Mode 1 (internal/external rotation), Mode 2 (flexion/extension), meniscal extrusion, and meniscal tears on the difference between MRI-based cartilage thickness and mJSW. The degree of underestimation and overestimation of ∆mJSW relative to ∆MRI-based cartilage thickness was determined using the β coefficients from the multivariable regression analysis in patients without meniscal extrusion or meniscal damage. Furthermore, the change in mJSW is often used as the surrogate measure of cartilage degeneration over a specific time. Therefore, our methodology was also applied to examine how changes between baseline and follow-up in Mode 1 and Mode 2 (∆Mode 1, ∆Mode 2), as well as meniscal extrusion and meniscal tears, influenced the change in mJSW compared to the change in MRI-based cartilage thickness (ΔDifference). Statistical significance was defined as a p-value < 0.05.

## 3 Results

### 3.1 Patients and data assessment

A total of 297 IMI-APPROACH patients Were initially considered for the study. However, 15 patients had missing MRI scan parameters (MOAKS and/or cartilage thickness), and 17 patients had incomplete, radiographic parameters (either ODIA software measurements or SSM were missing). As a result, a total of 265 patients were included in the first phase of the study. Every patient participated with one (index) knee. The characteristics of the patients included in the cross-sectional study are detailed in Table 1.

**Table 1:** The characteristics of the included IMI-APPROACH cohort patients.

	Baseline (N=265)
Age (years)	66.6 (7.2)
Sex, no. (%) women	206 (77.7%)
Body Mass Index (kg/m^2^)	27.9 (5.1)
mJSW medial compartment (mm)	3.6 (1.4)
Cartilage thickness medial compartment (mm)	3.5 (1.1)
Medial extrusion of the medial meniscus, no. (%)	
No meniscal extrusion	179 (67.5%)
Meniscal extrusion	79 (29.8%)
Unscorable	7 (2.6%)
Lateral extrusion of the lateral meniscus, no. (%)	
No meniscal extrusion	236 (89.1%)
Meniscal extrusion	27 (10.2%)
Unscorable	2 (0.8%)
K&L grade, no. (%)	
Grade 0	47 (17.7%)
Grade 1	72 (27.2%)
Grade 2	60 (22.6%)
Grade 3	72 (27.2%)
Grade 4 Missing	11 (4.2%) 3 (1.1%)

Mean (standard deviation (SD) are depicted, unless stated otherwise.; no., number; mJSW, minimum joint space width; K&L, Kellgren and Lawrence

The longitudinal analysis included 162 participants (77.8% women) with a baseline mean age of 66.0 years (SD 7.5) and BMI of 28.1 kg/m² (SD 5.2). The follow-up of the IMI-APPROACH patients occurred during the COVID-19 pandemic, which contributed to the missing follow-up data. Over 24 months, mean age increased by two years to 68.0 years, BMI remained stable at 27.9 kg/m², and mean mJSW of the medial compartment decreased from 3.8 mm (SD 1.3) to 3.7 mm (SD 1.4). The mean cartilage thickness of the medial compartment decreased from 3.0 mm (SD 0.7) to 2.9 mm (SD 0.7). Most participants remained within KL grades 2-4 (54%) at both baseline and 24 months follow-up. The absolute Δdifference between ΔMRI-cartilage thickness and ΔmJSW is 0.37 ± 0.36 mm. For patients with a 0 SD for rotation (mean rotation) (N = 24), this Δdifference between ΔMRI-cartilage thickness and ΔmJSW is 0.35 ± 0.37 mm. For patients with a 0 SD for flexion (mean knee flexion) (N = 29), this Δdifference is 0.5 ± 0.4 mm.

### 3.2 Statistical shape modes

**Fig. 2:**
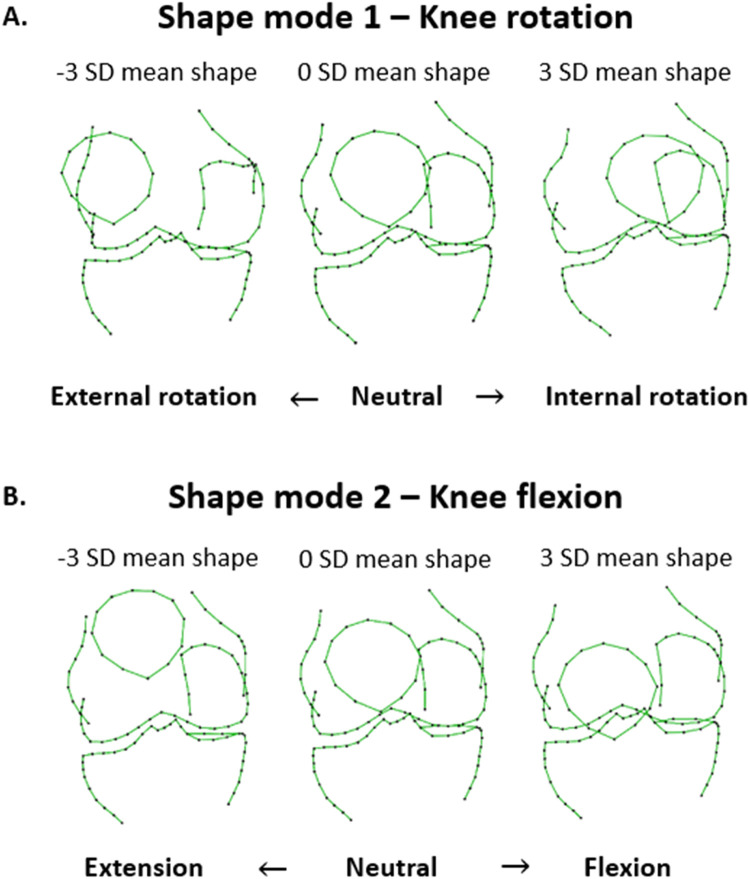
Positioning Mode 1 and Mode 2 of the IMI-APPROACH cohort. A) Shape mode 1 (knee rotation). The mean knee rotation (0 standard deviation (SD)) and the +3/-3 SD are shown. B) Shape mode 2 (knee flexion). The mean knee flexion (0 standard deviation (SD)) and the +3/-3 SD are shown.

24 shape modes were created to describe 90% of the shape variation. Shape mode 1, the primary mode of variation, represented knee rotation and accounted for 33.6% of total variation in shape. The positioning of both the patella and medial femoral condyles played a crucial role in discerning knee rotation (Fig. 2). A positive ΔMode 1 denoted an increase in internal rotation at follow-up (24 months) relative to baseline. Shape mode 2, reflecting knee flexion/extension accounts for 25.4% of total variation in shape. The location of the patella and medial tibial plateau indicated an important part of recognizing knee flexion (Fig. 2). A positive ΔMode2 denoted an increase in flexion.

### 3.3 Statistical shape modes and joint space width measurements

Univariable regression revealed that knee rotation (P = 0.01, β = -0.12) and flexion (P = 0.026, β = 0.11) significantly influenced cross-sectional differences between MRI cartilage thickness and mJSW. Medial (P = 0.002, β = 0.30) and lateral meniscal damage (P = 0.003, β = -0.36) were also significant, while meniscus extrusion showed no significant effects (Medial: P = 0.93, β = 0.00; Lateral: P = 0.65, β = -0.03).

**Fig. 3:**
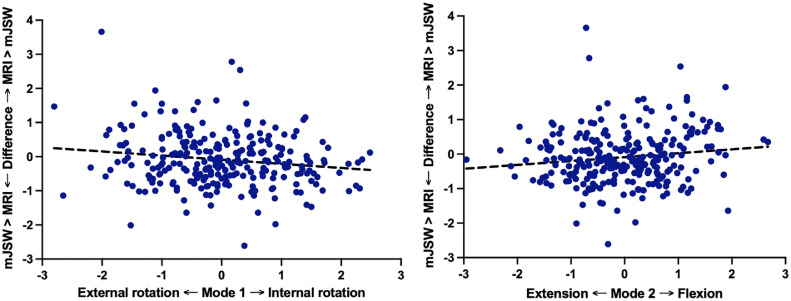
Relationship between the standard deviation of shape modes for rotation (A) and flexion/extension (B) and the difference between mJSW and cartilage thickness. The shape modes are represented by their standard deviations within this population, where 0 corresponds to the average shape mode (i.e., the average knee rotation and flexion/extension of the cohort). On the vertical axis the difference is provided by MRI-cartilage thickness – mJSW.

Using multivariable regression, knee rotation (mode 1) significantly influenced the cross-sectional difference between MRI cartilage thickness and mJSW (P < 0.001, β = -0.18) (Fig. 3). In Fig. 3, mode 1 is plotted against the difference between MRI cartilage thickness and mJSW. Internal rotation was associated with an overestimation of mJSW compared to MRI cartilage thickness (mJSW > MRI-based cartilage thickness), whereas external rotation caused an underestimation (mJSW < MRI-based cartilage thickness). In contrast, knee flexion (mode 2) showed no significant relationship with the cross-sectional difference (P = 0.20, β = 0.06) (Fig. 3). Medially extrusion of the medial meniscus and laterally extrusion of the lateral meniscus showed no significant relationship with the cross-sectional difference (Medial: P = 0.98, β = -0.00; Lateral: P = 0.91, β = 0.01). Finally, medial and lateral meniscal damage resulted in a significant difference between MRI cartilage thickness and mJSW (Medial: P < 0.001, β = 0.33; Lateral: P = 0.003, β = -0.38).

**Fig. 4:**
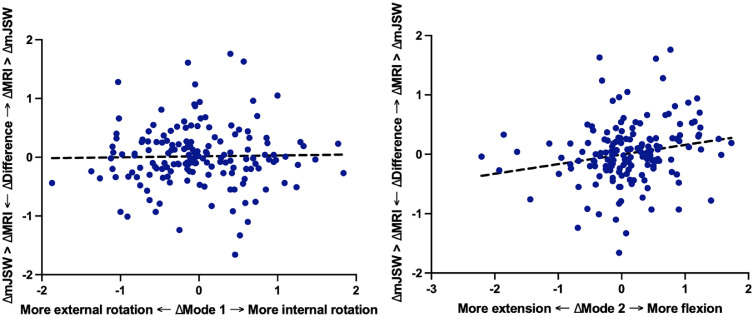
Relationship between differences in standard deviation of shape modes for rotation (A) and flexion/extension (B) and the Δdifference between changes of mJSW during follow-up and the changes in cartilage thickness found in MRI. The shape modes are represented by their standard deviations within this population, where 0 corresponds to the average shape mode (i.e., the average knee rotation and flexion/extension of the cohort). On the vertical axis the ∆difference is provided by ∆MRI-thickness -∆mJSW. Thus, a loss or gain in mJSW found during follow-up is skewed relative to the cartilage thickness measurements on MRI, dependent on the positioning differences of the knee during the two acquisitions of the radiographs.

In the multivariable analysis of longitudinal changes, more internal rotation at follow-up compared to baseline, indicated here as positive ∆Knee rotation (∆mode 1), showed no significant effects with the difference over time (P = 0.72, β = 0.02) (Fig. 4). In Fig. 4, ∆mode 1 is plotted against the ∆difference between MRI cartilage thickness and mJSW. ∆Knee flexion (∆mode 2) was significantly associated with longitudinal differences (P = 0.002, β = 0.19) (Fig. 4), where more knee flexion at follow-up results in a reduction in mJSW that does not represent an actual decrease in mJSW, while more extension at follow-up can create a false impression of mJSW increase. Medially extrusion of the medial meniscus and laterally extrusion of the lateral meniscus showed a significant relationship with the longitudinal difference (Medial: P < 0.001, β = 0.16; Lateral: P < 0.001, β = -0.21). Meniscal damage showed no significant associations with the longitudinal difference (Medial: P = 0.39, β = 0.07; Lateral: P = 0.08, β = -0.17).

We determined the degree of underestimation (mJSW < MRI-based cartilage thickness) and overestimation (mJSW > MRI-based cartilage thickness) of ∆mJSW compared to ∆MRI-based cartilage thickness for patients without meniscal extrusion and meniscal damage, as this represents the largest group in our study population. A one SD increase in internal rotation causes a 0.02 mm overestimation of mJSW compared to MRI-based cartilage thickness, while a similar increase in flexion results in a 0.2 mm underestimation. When both parameters increase by one SD, mJSW is underestimated by 0.2 mm. These effects become more pronounced with a two SD increase. A two SD increase in internal rotation and a two SD increase in knee extension result in an overestimation of mJSW by 0.43 mm.

## 4 Discussion

In this study, we investigated whether differences between surrogate cartilage thickness (mJSW) from knee radiographs and cartilage thickness from knee MRI could be (at least partially) attributed to positioning during radiographical acquisition. This was determined at a single time point (cross-sectional) and over a two-year follow-up period (longitudinal). Our findings showed that knee rotation (determined by SSM mode 1) significantly affected the cross-sectional difference between the mJSW and the cartilage thickness from MRI when meniscal status was not considered. It was shown that internal rotation caused an overestimation and external rotation an underestimation of mJSW compared to MRI cartilage thickness. Knee flexion (SSM mode 2) had also a significant effect on the cross-sectional difference when meniscal status was not considered, where knee flexion caused an underestimation and knee extension an overestimation of mJSW compared to MRI-cartilage thickness. When examining the multivariable model, we observed that meniscal damage and knee rotation have a significant effect on the difference between the mJSW and the cartilage thickness from MRI. Knee flexion and meniscal extrusion did not have a significant effect.

Longitudinally, knee rotation showed no significant effect on the ∆difference between ΔmJSW and ΔMRI cartilage thickness. Knee flexion differences (∆mode 2) showed a significant effect on the Δdifference. More specifically, knee flexion resulted in an underestimation, while knee extension led to an overestimation of changes in mJSW. Thus, increased flexion at follow-up may result in a false conclusion of cartilage loss based on mJSW, while increased extension may lead to undetected cartilage thinning or even a falsely detected increase in cartilage (surrogate) thickness. Meniscal extrusion at baseline showed a significant effect on the longitudinal differences, and meniscal damage showed no significant effect on the Δdifference. Notably, a 2 SD increase in internal rotation and a 2 SD increase in knee extension resulted in a false increase of mJSW by 0.43 mm. These findings underscore the critical role of knee positioning during radiographic acquisition in contributing to the differences between mJSW and MRI-based cartilage thickness.

Knee rotation and flexion biases are well-known limitations in measuring KOA progression using radiographs [12,13,[20], [21], [22]]. Studies have shown that variations in knee positioning can significantly affect JSW measurements, leading to inconsistencies in assessing cartilage degeneration. Kan et al*.* [12] found that medial JSW was significantly smaller in flexion view compared to the extended view, while Kinds et al*.* [13] concluded that leg extension increases JSW measurements. Our research demonstrates that these positioning effects may contribute to the differences between MRI-based cartilage thickness and radiographic mJSW measurements. Despite using the standardized Buckland-Wright protocol [3] in our study, the shape modes with the largest variation were those related to knee positioning, specifically rotation and flexion. We employed SSMs due to their ability to capture and quantify these variations in shape in a rigorous mathematical approach, expressing the variation in terms of standard deviation within the measured population. One of the key strengths of this method is its ability to make even small variations in knee positioning visible and measurable. In the study by Haverkamp et al*.* [26], SSMs were also used to assess the effect of knee flexion, concluding that OA knees were more extended compared with control knees. It is possible that the patient's positioning may be influenced by the thickness of the MRI cartilage and thus the severity of KOA. Additionally, patients experiencing high levels of pain may have limited knee extension. Ultimately, various factors could contribute to the positioning of the patient. In our study, we compare mJSW measurements with the reference, MRI-based cartilage thickness, and examined the effect of positioning over time, providing us more sensitive measurements per patients and offering a deeper understanding of how positioning influences the difference between cartilage thickness and mJSW.

In addition to the variability in knee positioning, we observed a considerable variability in the difference between the mJSW and MRI-based cartilage thickness, as revealed in Fig. 3, Fig. 4. The mJSW measurements are influenced by multiple factors, including the measurement technique, and the specific radiographic protocol used [35]. Notably, automatic measurement of mJSW resulted in more consistent outcomes than the manual measurement method, highlighting the potential benefits of automated techniques for improving measurement accuracy [36]. Previous research has extensively compared radiographic JSW measurements with cartilage thickness from non-weight-bearing MRI scans. While cross-sectional studies show strong correlations between these methods [14,15], longitudinal analyses often reveal weak or no significant correlations between changes in JSW and cartilage thickness [[16], [17], [18]], possibly due to factors such as meniscal extrusion, cartilage quality, loading effects, and positioning [19]. Meniscal extrusion does contribute to these differences over time. More specifically, the longitudinal difference between mJSW and MRI-based cartilage thickness was smaller in patients with medial or lateral meniscus extrusion compared to those without meniscal extrusion. However, it is important to note that most patients (89.1%) had no meniscal extrusion at baseline. Our study demonstrates that differences between cartilage thickness and mJSW can be partially explained by changes in radiographic positioning (knee rotation and flexion), with meniscal extrusion also contributing to these differences. This highlights the importance of considering positioning and technical aspects when interpreting mJSW measurements in both research and clinical settings.

Changes in mJSW serve as a primary outcome in numerous clinical investigations. Moreover, mJSW often serves as an inclusion criterion in OA studies, such as in the case of IMI-APPROACH cohort. In this cohort, mJSW was utilized as a parameter in a machine-learning model to predict the probability of increased or sustained knee pain and structural OA progression during the 24-month follow-up [27].

In addition to mJSW, other joint space width (JSW) measures exist, such as mean JSW and fixed location JSW [18,37]. However, mJSW is the most used measure in OA studies and clinical investigations. Our research highlights that, despite the standardized Buckland Wright radiograph acquisition protocol [3], knee rotation accounts for 33.6% and knee flexion for 25.4% of the total variation of 2D presented shape on the radiographs. It seems that there is a substantial influence of knee rotation and flexion on the truthfulness of classified progressors and non-progressors. This may result in falsely included patients into research cohorts based on their disease severity. Moreover, this method of mJSW analyses on radiographs may induce noise when categorizing patients into progressors and non-progressors in a research cohort, for instance for the assessment of treatment effectiveness. These insights emphasize the critical role of knee positioning during radiographical acquisition when mJSW is utilized as a primary endpoint or inclusion criterion in OA research. Even with the use of standardized radiograph acquisition protocols, we still observe the effect of knee positioning. Therefore, it is crucial to account for the influence of knee positioning when interpreting mJSW results. Looking ahead, it may be necessary to investigate alternative OA measures on radiographs that exhibit reduced sensitivity to positioning variations.

Our current study had several limitations. Firstly, we used cartilage thickness on MRI scans for comparison to mJSW. However, mJSW reflects both cartilage and meniscus and is measured in a weight-bearing position, whereas MRI-based cartilage thickness exclusively represents cartilage and is obtained in a non-weight-bearing position. Additionally, an increased cartilage thickness over time can be caused by cartilage swelling due to increased water content from collagen cleavage [[37], [38], [39], [40]]. However, this type of cartilage is likely more flexible and leads to a decrease in mJSW under loading such as occurs during the weight bearing radiographical acquisition, while simultaneously showing an increase in cartilage thickness on MRI performed without loading conditions [19]. In such case the MRI-based cartilage thickness does not serve as best representative of cartilage status and its ground truth becomes questionable. Secondly, potential measurement errors in mJSW measurements could influence the outcomes of this study. In most studies, mJSW is measured using specialized software such as e.g. Knee Imaging Digital Analysis [2]. However, we opted for fully automated software (ODIA) that automatically selects the anterior edge of both the lateral and medial tibial plateau and provides validated reproducible results [41] of mJSW over a specific region of the medial and lateral joint space. Thirdly, while our study primarily addresses the positioning of the patient’s knee in terms of flexion and rotation, it is important to note that the position of the X-ray tube above or below the knee joint can also affect mJSW measurements [22]. Fourthly, we only examined the effect of positioning on the differences in the medial compartment. This limitation means that potential effects on the lateral compartment were not assessed, which could provide a more comprehensive understanding of the impact of positioning on knee joint morphology. Fifthly, although this study demonstrates that positioning contributes to the differences between mJSW and actual cartilage thickness, the translation to clinically relevant differences remain an open question. This aspect requires further investigation in a larger study population to better understand how these variations impact clinical interpretation and decision-making. Moreover, our study does not clarify whether the change in mJSW due to positioning is explained by a different viewing angle of the joint or by an inherent change in JSW. This can be determined in future research. Finally, the timing during the day of the MRI-scan has the potential to impact cartilage thickness measurements. Both gravity and physical activity can affect cartilage thickness in the knee [42]. A study by Danieli et al*.* [42] demonstrated that after 60 minutes of running, cartilage thickness decreased significantly, ranging from 0.02 to 0.19 mm depending on the location.

In conclusion, our study highlights the significant impact of knee positioning on differences between weight-bearing mJSW and non-weight-bearing MRI cartilage thickness. Cross-sectional analyses revealed that knee rotation notably influenced these differences, with internal rotation overestimating and external rotation underestimating mJSW. Longitudinal analyses indicated that knee flexion played a role in longitudinal differences, where increased flexion at follow-up results in a reduction in mJSW that does not represent an actual decrease in mJSW. When both internal rotation and extension increased by 2 standard deviations from average in the current population, the combined effect resulted in a 0.4 mm false finding of surrogate cartilage change on radiographs. These findings underscore the critical role of knee positioning during radiographic acquisition in contributing to the differences between mJSW and MRI-based cartilage thickness.

## Ethical approval

All procedures performed in the IMI-Approach study were conducted in compliance with the protocol, Good Clinical Practice (GCP), the Declaration of Helsinki, and the applicable ethical and legal regulatory requirements (for all countries involved), and is registered under clinic altrials.gov identifier: NCT03883568. Informed consent was obtained from all individual participants included in the study.

## Funding

This work was supported by the Innovative Medicines Initiative Joint Undertaking under grant agreement no 115770, resources of which are composed of financial contribution from the European Union's Seventh Framework Programme (FP7/2007-2013) and EFPIA companies' in kind contribution. See www.imi.europa.eu and www.approachproject.eu.

## Declaration of competing interest

The authors declare the following financial interests/personal relationships which may be considered as potential competing interests:

Roel J.H. Custers: Received research grants from the Dutch Arthritis Association, Hy2Care, JointSphere, and ZonMw, all within the context of RegMedXB. All payments were made to the employer. Serves as Chair of the Data Safety Monitoring Board (DSMB) for the AIR study; compensation for this role was paid to the employer. Holds an unpaid leadership position as Chair of the working group “Joint Preserving Treatments & Regenerative Medicine” within the Dutch Orthopaedic Society.

Claudia Lindner: Received support for the present manuscript through a fellowship from the Wellcome Trust & Royal Society, UK (Grant number: 223267/Z/21/Z). Holds a pending patent titled *Image processing apparatus and method for fitting a deformable shape model to an image using random forest regression voting*. Royalties to be received if the patent is licensed.

Timothy Cootes: Received support for the present manuscript through grant funding provided to the institution by the EPSRC (UK), MRC (UK), and the Wellcome Trust. The algorithm for automatically locating points (*Random Forest Regression Voting Constrained Local Model*) is patented by the University of Manchester. A portion of licence fees will be received if the patent is licensed.

Francisco J Blanco: Received grants or contracts for clinical trials from Abbvie, Bristol Myers Squibb, Roche, Servier, Novartis, Horizon Therapeutics Ireland DAC, ITF RESEARCH PHARMA S.L.U., and GSK Research, with all payments made to the institution. Additional clinical trial funding received from Pfizer, Sanofi-Aventis, Grunenthal, Lilly, Merck Healthcare KGaA, and LG Chem, Ltd., as well as from UCB, Janssen, Amgen, Regeneron, Alkem Laboratories Ltd., Grünenthal, Sun Pharma Global FZE, and Kiniksa Pharmaceuticals GmbH; all payments were made to the institution. Received personal payments from Medicamenta-Ecuador, Grunenthal, and Asofarma for lectures, presentations, or educational events. Support for attending meetings and/or travel provided by UCB, Abbvie, and Celgene. Participates on an advisory board for Grunenthal, with payment made directly.

Simon C Mastbergen: Research is financially supported by the Dutch Arthritis Society (LLP9).

Wolfgang Wirth: Part-time employee at Chondrometrics GmbH. Received research grants from Horizon Europe (PROTO project) and Eurostars (OA-BIO project), with payments made to the institution. Engaged in image analysis contracts with the following institutions and companies, with all payments made to the institution: TissueGene (USA), Novartis AG (Switzerland), University of Basel (Switzerland), University of Sydney (Australia), Erlangen University (Germany), Peptinov (France), ICM (South Korea), Foundation for the NIH (two contracts), and the University of Western Ontario. Holds shares in Chondrometrics GmbH.

Harrie Weinans: Support for the present manuscript was provided through the APPROACH project (IMI grant via UMCU). Additional grants or contracts received via UMCU, not directly related to the current manuscript, include funding from Interreg (EFRO), OA-Inject (NWO), 3DHip (Eurostars), Dartbac (NWO), LUMINATE (EU), SHIELD (Eurostars), and Kansen voor West (Province of Utrecht). Holds minority shareholder positions in Replasia, Presurgeo, Amation, and Preimure.

Felix Eckstein: Support for the present manuscript was provided through the IMI EU APPROACH project via the University of Utrecht, with payment made as a subcontract to Chondrometrics. Additional grants or contracts were received by Chondrometrics from the University of Erlangen, Kolon TissueGene, Galapagos/Servier, Novartis, and the EU Eurostars program. Received consulting fees directly from Kolon TissueGene, Galapagos, Novartis, 4P Pharma, Formation Bio, Peptinov, Artialis, and Sanofi. Serves on advisory boards or Data Safety Monitoring Boards for Galapagos, 4P Pharma, and Formation Bio (TrialSpark). Majority owner of Chondrometrics GmbH.

Frank Roemer: Received grants or contracts from the Else Kröner-Fresenius Stiftung, with payments made to the institution. Received consulting fees from Grünenthal GmbH, paid directly. Shareholder in Boston Imaging Core Lab (BICL), LLC, with payments made directly. Serves as Editor in Chief of the *Osteoarthritis Imaging* journal.

Francis Berenbaum: Received consulting fees from Grunenthal, GSK, Eli Lilly, Novartis, Pfizer, Servier, 4P Pharma, and Peptinov. Received payment or honoraria for lectures, presentations, speakers bureaus, manuscript writing, or educational events from Viatris, Pfizer, and Zoetis. Support for attending meetings and/or travel was provided by Nordic Pharma. Holds patents planned, issued, or pending with 4Moving Biotech. Participates on Data Safety Monitoring Boards or Advisory Boards for AstraZeneca, Sun Pharma, and Nordic Bioscience. Holds stock or stock options in 4P Pharma and 4Moving Biotech.

Ida Haugen: Received consulting fees from Novartis, GSK, and Grünenthal, with payments made directly to self. Received payment or honoraria for lectures, presentations, speakers bureaus, manuscript writing, or educational events from Abbvie, paid directly to self.

Margreet Kloppenburg: Received grants or contracts from IMI APPROACH and the Dutch Arthritis Society, with all payments made to the institution. Consulting fees for advisory board participation received from Pfizer, UCB, CHDR, GSK, Novartis, and Peptinov, paid to the institution. Payment or honoraria for lectures, presentations, speakers bureaus, manuscript writing, or educational events received from Novartis, paid to the institution. Held leadership or fiduciary roles as a member of the OARSI board (2017–2022), member of the EULAR council (chair of the advocacy committee), and President of the Dutch Society for Rheumatology.

Francis Berenbaum: Received consulting fees from Grunenthal, GSK, Eli Lilly, Novartis, Pfizer, Servier, 4P Pharma, and Peptinov. Received payment or honoraria for lectures, presentations, speakers bureaus, manuscript writing, or educational events from Viatris, Pfizer, and Zoetis. Support for attending meetings and/or travel was provided by Nordic Pharma. Holds patents planned, issued, or pending with 4Moving Biotech. Participates on Data Safety Monitoring Boards or Advisory Boards for AstraZeneca, Sun Pharma, and Nordic Bioscience. Holds stock or stock options in 4P Pharma and 4Moving Biotech.

Ida Haugen: Received consulting fees from Novartis, GSK, and Grünenthal, with payments made directly to self. Received payment or honoraria for lectures, presentations, speakers bureaus, manuscript writing, or educational events from Abbvie, paid directly to self.

Margreet Kloppenburg: Received grants or contracts from IMI APPROACH and the Dutch Arthritis Society, with all payments made to the institution. Consulting fees for advisory board participation received from Pfizer, UCB, CHDR, GSK, Novartis, and Peptinov, paid to the institution. Payment or honoraria for lectures, presentations, speakers bureaus, manuscript writing, or educational events received from Novartis, paid to the institution. Held leadership or fiduciary roles as a member of the OARSI board (2017–2022), member of the EULAR council (chair of the advocacy committee), and President of the Dutch Society for Rheumatology.
